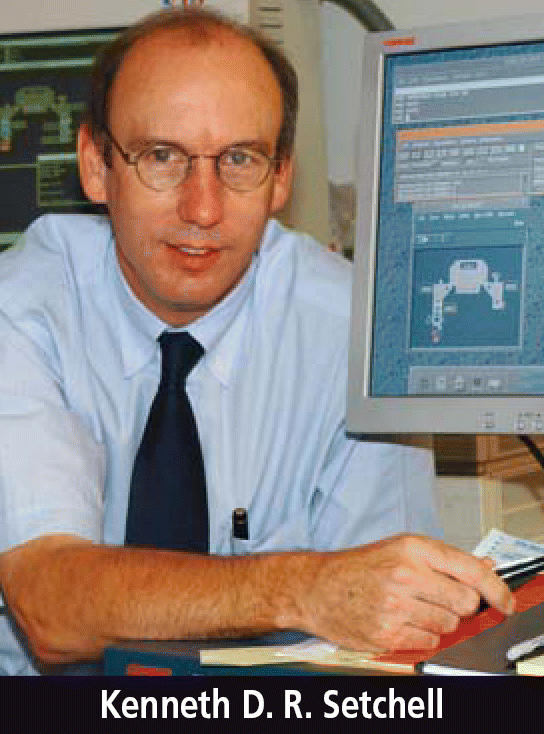# Guest Editorial: Assessing Risks and Benefits of Genistein and Soy

**DOI:** 10.1289/ehp.114-a332

**Published:** 2006-06

**Authors:** Kenneth D.R. Setchell

**Affiliations:** Department of Pathology, Cincinnati Children’s Hospital Medical Center, University of Cincinnati College of Medicine, Cincinnati, Ohio E-mail: Kenneth.Setchell@cchmc.org

Yet again, the humble soybean has undergone a further cycle of critical scrutiny. The extent to which soy foods and products—or, more relevantly, one of the constituent bioactive components, genistein—pose potential health benefits or risks has once again come under the microscope. Hot on the heels of the American Heart Association’s conclusions that soy has little effect on reducing cholesterol or improving other cardiovascular risk markers (Sacks et al. 2006), there has been yet a further review of the safety of genistein and soy infant formula, this time by a panel of 14 independent “experts” convened by the National Toxicology Program’s Center for the Evaluation of Risks to Human Reproduction (NTP-CERHR 2006a, 2006b). Two separate draft reports comprehensively document an extensive literature published on the reproductive and developmental toxicity of genistein, and on soy infant formula, respectively. Following a public forum held in Washington, DC, in March 2006, these reports concluded that there is negligible concern for genistein in the general adult population, although there are insufficient data to permit conclusions for soy formula. Given 40 years’ use of soy formula in an estimated 25–30 million infants, this paucity of information in the scientific literature could and should be taken as compelling evidence of the assurance of the safety of soy infant formula. Certainly if soy infant formula were a “drug entity,” with this track record its safety would never be in question. So why, then, has there been so much controversy over soy’s safety? After all, the soybean is a vegetable protein of the highest quality, has no cholesterol, is high in unsaturated fats, is a good source of fiber and complex carbohydrates, and is free of lactose. It is a food consumed daily by millions of adults and children in Asia, where the incidence of hormone-dependent diseases is relatively low (although now increasing) compared with countries where soy is not typically consumed. Central to this issue is the fact that the soybean is the champion plant species in delivering a dietary source of isoflavones, an important class of phytoestrogen.

Genistein is clearly a bioactive molecule. It displays characteristics of a selective estrogen receptor (ER) modulator (SERM) rather than an estrogen (Pike et al. 1999), showing affinity for ERβ (Kuiper et al. 1998), and this distinction has implications for its potential actions. It has many nonhormonal activities relevant to potential effects at the cellular and molecular level. However, genistein, with few exceptions, is not a major isoflavone of most soy foods and products consumed in Western countries, unless these have undergone fermentation, as in traditional foods such as tempeh, natto, and to some extent miso, consumed mainly by Asians (Coward et al. 1993). It accounts for < 2% of the isoflavone content of the soybean, soy proteins, and most Western soy foods, including soy infant formulas. Although innumerable studies in animal models, mostly rodents, clearly show that purified genistein can induce adverse reproductive effects, most of these findings have little relevance to humans consuming soy foods, and especially to infants fed soy formulas.

In elucidating the safety and toxicity of isoflavones in soy foods, one might ask why genistin and daidzin have not been tested. After all, these are the major isoflavones of soybeans and most soy foods, and these compounds are commercially available. The answer is simple: These sugar conjugates would be largely devoid of *in vitro* activity, and *in vivo* would have no overt reproductive toxicity, even though biologic responses would be expected at the cellular and molecular level. Soy meal is a key ingredient of most commercial rodent diets routinely used by animal breeding establishments and research institutes, and rats and mice are exposed to doses ranging 80–160 mg/kg bw of total isoflavones, higher than doses of purified genistein injected in many toxicologic rodent experiments revealing adverse events (Brown and Setchell 2001; Thigpen et al. 2004). Furthermore, soy meal is routinely fed to domestic farm animals as an important source of protein, with no apparent reproductive toxicity. As a point of reference, exposure via soy foods is 0.5–1.5 mg/kg body weight for *total* isoflavones in adults, of which genistein accounts for only 0.005–0.015 mg/kg body weight, whereas exposures are an order of magnitude higher in infants fed soy formulas (Setchell et al. 1997, 1998).

Route of administration, animal model, species differences, and metabolism are the most crucial factors in considering the effects of genistein. The mouse, rat, and monkey metabolize soy isoflavones differently from human adults and infants, producing almost exclusively equol (Setchell et al. 2002, 2005). Little genistein circulates after feeding soy to rodents, so why is genistein used to predict isoflavone exposure from soy in this species? Indeed, emerging microarray and molecular studies comparing genistein with soy show just how differently these two entities behave (Ronas et al. 2006); and this holds true for genistein and estradiol (Diel et al. 2000). Genistein behaves differently when injected versus given orally, and herein lies the problem in making extrapolations. The intestine provides a key barrier to limiting the bioavailability and biologic activity of isoflavones administered orally. Delivery of genistein by injection bypasses first-pass metabolism and leads to plasma concentrations of free genistein that are far higher than if administered orally, particularly at high doses. Finding isoflavone concentrations in rodents given genistein to be comparable to those of infants fed soy formula is of little relevance when route of administration differs. If a drug is to be given orally, the Food and Drug Administration requires assessment of safety/toxicity by the oral route, and this should be the rule for isoflavones when designing studies evaluating the risks of soy foods. Finally, although the prenatal/neonatal rodent has proven an appropriate model for *in utero* human exposure to an endocrine disruptor, as illustrated in excellent studies of diethyl-stilbestrol (McLachlan et al. 1980, 2001; Newbold 2004; Yoshida et al. 1999), it is of little value for assessing postnatal development of infants exposed to isoflavones from soy formula. The only appropriate model for postnatal human reproductive development is the human infant.

If a drug is to be given orally, the Food and Drug Administration requires assessment of safety/toxicity by the oral route, and this should be the rule for isoflavones when designing studies evaluating the risks of soy foods.

Confidence in the data from clinical studies has been shaken by the high variability in the findings from clinical studies of soy protein and soy foods. Although not at all surprising, given the lack of consistency among study designs, this has diminished consumer confidence for soy as a healthy food option. A legitimate area of concern is soy use in Western women diagnosed with breast cancer and in those at high risk for breast cancer. Recommendations for the former are difficult to make at this time, but encouraging findings for the prophylactic effect of the SERM raloxifene on breast cancer may offer some promise for soy in the latter group (Cummings et al. 1999; Martino et al. 2004, 2005). To expect that soy and its constituent isoflavones will reverse or arrest chronic disease is asking too much of this small bean. The greatest potential for soy lies not in using it to treat pathologic changes that are usually irreversible, but in including it in the diet early in life, which will, by whatever mechanism, offer the potential for preventing chronic diseases. What is needed is a move toward prospective studies to demonstrate the risk/benefit of soy and its bioactive constituents, whether isoflavones, protein, or other components, rather than more animal studies that will unquestionably show many of the same effects already well documented. Might there be long-term health benefits from early feeding of soy formula or soy foods to children? Until such studies are executed and data available, there will be no resolution on this issue, and we face the prospect of throwing the baby out with the bathwater. Common sense should prevail.

## Figures and Tables

**Figure:**